# Dissecting the Roles of Supervised and Unsupervised Learning in Perceptual Discrimination Judgments

**DOI:** 10.1523/JNEUROSCI.0757-20.2020

**Published:** 2021-01-27

**Authors:** Yonatan Loewenstein, Ofri Raviv, Merav Ahissar

**Affiliations:** ^1^The Edmond and Lily Safra Center for Brain Sciences; ^2^Department of Neurobiology, The Alexander Silberman Institute of Life Sciences; ^3^Department of Cognitive Sciences; ^4^The Federmann Center for the Study of Rationality; ^5^Department of Psychology, The Hebrew University of Jerusalem, Jerusalem, Israel 9190401

**Keywords:** contraction bias, frequency discrimination, perception, supervised learning, unsupervised learning

## Abstract

Our ability to compare sensory stimuli is a fundamental cognitive function, which is known to be affected by two biases: choice bias, which reflects a preference for a given response, and contraction bias, which reflects a tendency to perceive stimuli as similar to previous ones. To test whether both reflect supervised processes, we designed feedback protocols aimed to modify them and tested them in human participants. Choice bias was readily modifiable. However, contraction bias was not. To compare these results to those predicted from an optimal supervised process, we studied a noise-matched optimal linear discriminator (Perceptron). In this model, both biases were substantially modified, indicating that the “resilience” of contraction bias to feedback does not maximize performance. These results suggest that perceptual discrimination is a hierarchical, two-stage process. In the first, stimulus statistics are learned and integrated with representations in an unsupervised process that is impenetrable to external feedback. In the second, a binary judgment, learned in a supervised way, is applied to the combined percept.

**SIGNIFICANCE STATEMENT** The seemingly effortless process of inferring physical reality from the sensory input is highly influenced by previous knowledge, leading to perceptual biases. Two common ones are contraction bias (the tendency to perceive stimuli as similar to previous ones) and choice bias (the tendency to prefer a specific response). Combining human psychophysical experiments with computational modeling we show that they reflect two different learning processes. Contraction bias reflects unsupervised learning of stimuli statistics, whereas choice bias results from supervised or reinforcement learning. This dissociation reveals a hierarchical, two-stage process. The first, where stimuli statistics are learned and integrated with representations, is unsupervised. The second, where a binary judgment is applied to the combined percept, is learned in a supervised way.

## Introduction

**Figure 1.**
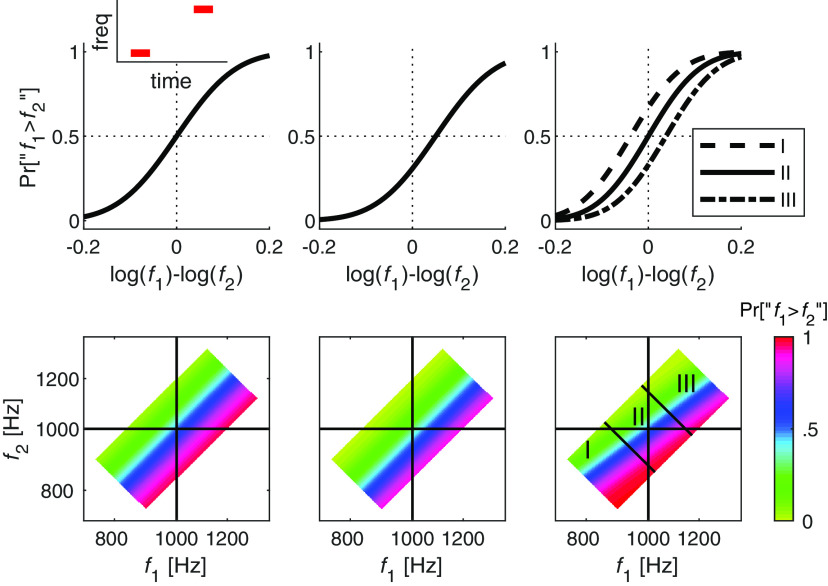
Choice and contraction biases in the delayed-comparison task. Top, left, inset, A schematic illustration of the task, in which a participant is presented with two, temporally-separated, pure tones and is instructed to report which one is larger. Top, Schematic illustrations of the psychometric curves, the probability of responding that the frequency of the first stimulus was greater than that of the second stimulus, “$f_{1}>f_{2}$”, as a function of the frequency difference between the two stimuli in logarithmic scale. Bottom, Schematic illustrations of the same analyses in the $f_{1}\times f_{2}$ plane (in logarithmic scale). Color code denotes the probability of responding “$f_{1}>f_{2}$”. Left, An unbiased participant. The psychometric function is centered around a zero frequency difference between the stimuli, and the line of indifference (blue) overlaps the diagonal. Middle, Choice bias manifests as a horizontal shift (here rightwards) of the psychometric curve. Right, Contraction bias. When two tones are relatively low (Region I), the first tone is contracted to a higher value, yielding a tendency to respond “$f_{1}>f_{2}$", and to a leftward shift of the psychometric curve (dashed line). When the tones are relatively high (Region III), the psychometric curve is shifted to the right (dashed-dotted line). The psychometric curve is unbiased (solid line) only in the intermediate region (II). In the $f_{1}\times f_{2}$ plane (in logarithmic scale), the contraction bias manifests as a line of indifference whose slope is <1. The colored region denotes the pairs of stimuli used in the experiments.

Perceptual discrimination, the ability to compare sensory stimuli, is a fundamental cognitive function, which has been extensively studied using the delayed-comparison task. In this paradigm, the participant (human or animal) is presented with two temporally separated stimuli that differ along a single dimension, e.g., pitch, intensity, luminance, or contrast, and is instructed to report which one is “larger” along that dimension (e.g., frequency; Fig. 1, inset). The standard way of quantifying performance in this task is the psychometric curve, which depicts the probability that the participant would report that the first stimulus is larger than the second as a function of the difference between the two stimuli. The slope of the psychometric function is often interpreted as reflecting the level of internal noise that limits perceptual resolution. However, this slope does not capture two common biases, choice bias and contraction bias.

Choice bias (also known as the stationary response bias; Jones, et al., 2015) is the tendency to prefer a specific response (Green and Swets, 1966; Klein, 2001; Lebovich et al., 2019). It has been shown that choice bias is sensitive to feedback (Herzog and Fahle, 1999): responses that have more often been associated with a “correct-answer” feedback are more likely to be preferred (Gold and Ding, 2013). Typically, such sensitivity to feedback improves performance because it allows the participant to exploit associations between actions and their outcome (Shteingart and Loewenstein, 2014).

Contraction bias is the tendency to perceive stimuli as closer to the “center” of the distribution of similar, previously-presented, stimuli (also known as “central tendency”; Hollingworth, 1910; Poulton, 1989). Typically, it biases perception toward a more probable interpretation of the sensory input. It has been hypothesized that the contraction bias is the consequence of incorporating stimuli-specific expectations into perception to increase perceptual accuracy (Huttenlocher et al., 2000). This hypothesis leads to two predictions. Rather than reflecting a rigid biophysical property of the sensory system, (1) contraction bias is sensitive to the statistical distribution of the stimuli used in the experiment; (2) contraction bias increases as the reliability of stimulus representation decreases. Both predictions have been verified (Huttenlocher et al., 2000; Ashourian and Loewenstein, 2011; Lieder et al., 2019).

We used a Perceptron (a linear discriminator) to model decision-making in the delayed-discrimination task. Perceptual comparison in the Perceptron is a two-stage process. In the first stage, the representations of the two stimuli are linearly combined, and in the second stage, a binary decision is made. Both contraction and choice biases can be directly mapped to the two parameters determining the Perceptron's first and second stages of computation, respectively. Therefore, an optimal Perceptron, a Perceptron whose two parameters are tuned to maximize “correct-response” feedback, specifies the optimal values of these two biases, within a given feedback protocol, and can be used to compare human behavior to optimal performance.

We found that when feedback is unbiased, the optimal Perceptron model provides a better fit to participants' behavior than the psychometric curve, indicating a similarity between human and optimal performances. This optimality could result from supervised learning, a term used in Machine Learning to indicate situations in which the learner utilizes information during the training procedure (experience) that it lacks during the testing phase (Shalev-Shwartz and Ben-David, 2014). In delayed discrimination, this information is the “correct-answer” feedback. Human near optimality could reflect the use of the feedback to maximize the fraction of correct-responses. Choice bias sensitivity to feedback can be explained in this framework. If contraction bias also reflects supervised learning, it should also be sensitive to the feedback protocol. Alternatively, it could result from statistical learning of stimuli's distribution (unsupervised learning), and thus be feedback insensitive. We found that in contrast to choice bias, contraction bias is insensitive to the feedback, indicating that in this task, feedback has access only to the decision stage and not to the earlier stage, whose parameters are learned in an unsupervised way.

## Materials and Methods

### 

#### The delayed comparison task

Participants were instructed to compare two serially presented tones and to indicate which of the two stimuli (first or second) had a higher pitch (Fig. 1, inset). Performance in discrimination tasks is typically depicted by the psychometric curve plotted in top row of Figure 1. A fuller, albeit less conventional representation of participants' responses on such tasks, is presented in Figure 1, bottom row. This two-dimensional representation reflects the probability of this response for each pair of stimuli: the axes are the frequencies of two stimuli in logarithmic scale (log f1×log f2), and the participants' probability of response Pr [“$f_{1}>f_{2}$”] is color coded. The left plot illustrates the predicted responses of an unbiased participant. The probability of the response depends solely on the difference between the two stimuli (log f1-log f2), and therefore, the probability of the response does not change when moving in parallel to the diagonal in the log f1×log f2 plane, in which log-frequency difference does not change. In the absence of a choice bias, the line of indifference, in which Pr[“$f_{1}>f_{2}$”] = 0, lies along this diagonal.

Choice bias is manifested as a lateral shift of the psychometric curve: a rightward shift of the curve (Fig. 1, top, middle) reflects a tendency to respond that the first stimulus was smaller than the second, whereas a leftward shift reflects the opposite bias. In the log f1×log f2 representation (Fig. 1, bottom, middle), this shift is manifested as a shift in the line of indifference to the right (preferring the second stimulus; Fig. 1, bottom, middle) or to the left (preferring the first; not shown) without changing its slope. In both these cases, the conventional psychometric plot captures participants' behavior because the responses are determined solely by the difference between the two stimuli, log f1-log f2. However, this is not true in the case of contraction bias, which cannot be depicted by a single psychometric curve, as explained below.

As discussed in the Introduction, the magnitude of the contraction bias increases with the noise/uncertainty in the representation of the stimulus. In the context of delayed-discrimination tasks, the representation of the first stimulus is noisier than that of the second stimulus by the time the decision is made. This is because encoding and retaining the first stimulus in memory degrades its representation. Consequently, the contraction of the first stimulus to the center of the distribution is larger than the contraction of the second stimulus (Berlineret al., 1977; Preuschhofet al., 2010; Ashourian and Loewenstein, 2011; Raviv et al., 2012, 2014). When the stimuli are smaller than the median, the contraction bias favors responding “$f_{1}>f_{2}$”, thus shifting the psychometric curve leftwards. When they are larger than the median, contraction bias shifts the psychometric curve rightward (Fig. 1, top, right). In the log f1×log f2 space, it changes the slope of the line of indifference, making smaller than 1 (Fig. 1, bottom, right). The stronger the contraction bias, the smaller the slope of the line of indifference. Infinite contraction would manifest as a horizontal indifference line, in which participants' responses are fully determined by the second stimulus.

#### The stimuli

Each participant performed 220 trials. Each trial consisted of two 50-ms pure tones, with a 10-ms linear rise time, and a 10-ms linear fall time, separated by a 950-ms interstimulus interval. Immediately after the second stimulus was played, the text “Which tone was higher?” appeared on screen, and the participant responded by clicking one of two on-screen buttons using a computer mouse, with no time constraint. Visual feedback of a smiling face or a sad face was presented for 300 ms after correct and incorrect responses, respectively. After a pause of 700 ms, the next trial began. The frequencies of the two tones (in Hz), $f_{1}$ and $f_{2}$ were chosen such that log f1+log f2/2 was uniformly distributed between $log\left(1000\right)-0.2$ and $log\left(1000\right)+0.2$, where log denotes natural logarithm. In 75%, randomly selected trials, log f1-log f2/2 was uniformly distributed between -0.0905 and 0.0905. This resulted in a uniform distribution of stimuli (in logarithmic scale) in the rectangles in Figure 1, bottom. The feedback in these trials was always correct. In the remaining 25% of the trials, which were denoted “impossible” trials, the frequency of $f_{1}$ was equal to that of $f_{2}$ ($f_{1}=f_{2}$). The feedback in these trials varied according to the experimental condition.

#### Participants

This study was approved by the Hebrew University Committee for the Use of Human Subjects in Research. A total of 200 adult participants of either sex were recruited using the online labor market Amazon Mechanical Turk.

In order to verify that participants understood the task correctly, and paid full attention for the whole duration of the block, we excluded blocks from the analysis in which performance in the first half (110 trials) of the block, or the second half of the block did not differ significantly (p<0.05) from chance level performance. On average, this translated to a requirement of at least 62% correct responses on the possible trials of each of the two halves of the block; however, the exact criterion changed from block to block, depending on the number of possible trials. Together, 30/200 of the participants were excluded from the analysis.

#### The Perceptron model and Bayesian inference

We consider the case in which as in Equation 1, $r_{1}=s_{1}+n$; $r_{2}=s_{2}$, such that $n∼N\left(0,\sigma^{2}\right)$. We further assume that $s_{1}∼N\left(0,\Sigma^{2}\right)$. To infer $s_{1}$ from $r_{1}$, we use Bayes' rule:

$Pr\left[s_{1}|r_{1}\right]\propto Pr\left[r_{1}|s_{1}\right]\cdot Pr\left[s_{1}\right]\propto e^{-\frac{{\left(s_{1}{-r}_{1}\right)}^{2}}{2\sigma^{2}}}\cdot e^{-\frac{{\left(s_{1}\right)}^{2}}{2\Sigma^{2}}}\propto e^{-\frac{{\left(s_{1}-\mu \right)}^{2}}{2\rho^{2}}}$ where $\mu =\frac{1}{1+\frac{\sigma^{2}}{\Sigma^{2}}}\cdot r_{1}$ and $\rho^{2}=\frac{1}{\frac{1}{\sigma^{2}}+\frac{1}{\Sigma^{2}}}$. Therefore, given $r_{1}$ and $s_{2}$: $Pr\left[s_{1}>s_{2}|r_{1},s_{2}\right]=\int_{s_{2}}^{\infty }\frac{dz}{\sqrt{2\pi \rho^{2}}}e^{-\frac{{\left(z-\mu \right)}^{2}}{2\rho^{2}}}$. The policy that maximizes performance is thus to report that $s_{1}>s_{2}$ if and only if $\frac{1}{1+\frac{\sigma^{2}}{\Sigma^{2}}}\cdot r_{1}>s_{2}$, which can be implemented in a Perceptron with $a=\frac{1}{1+\frac{\sigma^{2}}{\Sigma^{2}}}$ and b=0.

## Results

### The impact of feedback protocols on human performance

#### Feedback protocols

Though both the choice and contraction biases are well documented in humans and other animals, the processes that affect them are only partly understood. In particular, it remains unclear whether the contraction bias can be modified by the feedback protocol (where we used a smiling/a sad face to indicate a correct/incorrect response). To explore this issue, we used a pitch discrimination task, where tone frequency determines the perceived pitch (Fig. 1). We designed five different feedback schedules and administered them to five different groups of participants: one was administered as a control, two were designed to enhance choice biases, namely, the fraction of “$f_{1}>f_{2}$” or “$f_{2}>f_{1}$” responses, and two aimed to enhance or reduce contraction bias. In order to manipulate the biases without providing false feedback, we incorporated 25% “impossible trials,” namely, trials in which the two tones had the same frequency ($f_{1}=f_{2}$). Feedback was only biased in these impossible trials. In the control protocol, feedback on the impossible trials was random, with equal probabilities for the two possible responses being considered “correct.” In the protocol designed to enhance the “$f_{1}>f_{2}$” response, the “$f_{1}>f_{2}$” response was considered “correct” in 90% of the impossible trials, whereas in the protocol aimed to enhance the “$f_{2}>f_{1}$” responses, it was considered “correct” only in 10% of the impossible trials. In the protocol aimed to enhance contraction bias, responses congruent with the contraction bias ($f_{1}>f_{2}$ when the two tones were below the median and $f_{1}<f_{2}$ when they were above the median) were considered “correct” in 90% of the impossible trials. These responses were considered “correct” only in 10% of the trials in the protocol aimed to suppress the contraction bias. The feedback on all possible trials, i.e., the remaining 75% of the trials, was veridical and did not differ between groups (Table 1).

**Table 1.** Feedback protocols

		*f*_1_ = *f*_2_ < 1*kHz*	*f*_1_ = *f*_2_ > 1*kHz*
Control	Unbiased	*f*_1_ > *f*_2_, 50%	*f*_1_ > *f*_2_, 50%
		*f*_1_ < *f*_2_, 50%	*f*_1_ < *f*_2_, 50%
Choice bias	Enhance *f*_1_ > *f*_2_	*f*_1_ > *f*_2_, 90%	*f*_1_ > *f*_2_, 90%
		*f*_1_ < *f*_2_, 10%	*f*_1_ < *f*_2_, 10%
	Suppress *f*_1_ > *f*_2_	*f*_1_ > *f*_2_, 10%	*f*_1_ > *f*_2_, 10%
		*f*_1_ < *f*_2_, 90%	*f*_1_ < *f*_2_, 90%
Contraction bias	Enhance contraction bias	*f*_1_ > *f*_2_, 90%	*f*_1_ > *f*_2_, 10%
		*f*_1_ < *f*_2_, 10%	*f*_1_ < *f*_2_, 90%
	Suppress contraction bias	*f*_1_ > *f*_2_, 10%	*f*_1_ > *f*_2_, 90%
		*f*_1_ < *f*_2_, 90%	*f*_1_ < *f*_2_, 10%

Feedback in the impossible trials depended on the values of $f_{1}=f_{2}$ relative to the median of the distribution (1 kHz) and on the protocol type. The third and fourth column denote the probabilities that the two responses were considered “correct.” Feedback in the “possible” trials reflected the veridical difference between the two stimuli.

#### Feedback modifies choice bias

**Figure 2.**
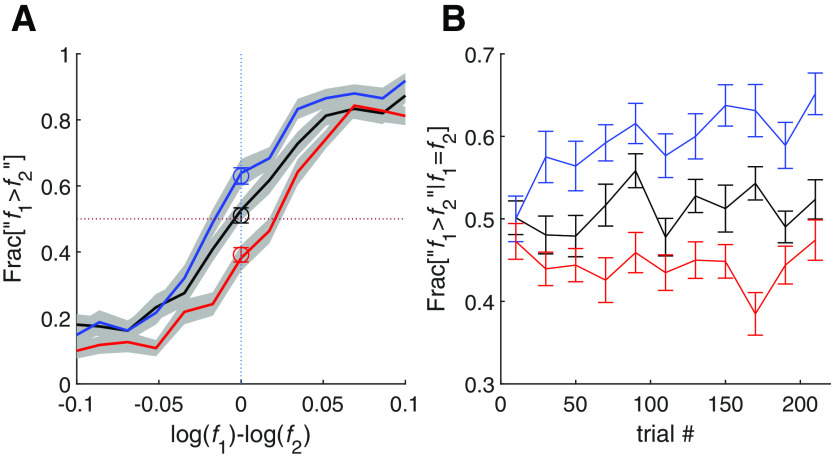
Choice bias is quickly modified by the feedback protocol. ***A***, The psychometric curves of the three feedback groups, control (black); feedback favoring “$f_{1}>f_{2}$” response (blue); feedback favoring “$f_{2}>f_{1}$” (red). Gray areas around the curves denote the cross-participant SEM. Open symbols denote responses on impossible trials. The psychometric curves were constructed using only the possible trials. ***B***, Dynamics of responding “$f_{1}>f_{2}$” on impossible trials in each of the three groups. Each dot is average over 20 trials (approximately five impossible trials) and all participants. Whereas the three groups all started with no choice bias, they deviated according to the rewarded response within <40 trials. Error bars denote cross-participant SEM.

Figure 2*A* depicts the psychometric curves of three groups of participants associated with the different feedback protocols: the control (black), favoring the response “$f_{1}>f_{2}$” (blue), and favoring the response “$f_{2}>f_{1}$” (red). Whereas there was no consistent choice bias in the control group, the other two groups exhibited substantial choice biases, which were manifested as shifted psychometric curves.

To further illustrate the effect of the feedback protocol on the choice bias, Figure 2*B* depicts the proportion of participants responding “$f_{1}>f_{2}$” in the impossible trials in each of the three groups as a function of trial number. At the beginning of the assessment, this proportion was similar in the three groups. However, it quickly deviated, such that within fewer than 40 trials the two groups with opposing feedback differed significantly in their choice bias (p < 0.02,one-sided Wilcoxon rank-sum test over participants).

#### Feedback does not affect contraction bias

**Figure 3.**
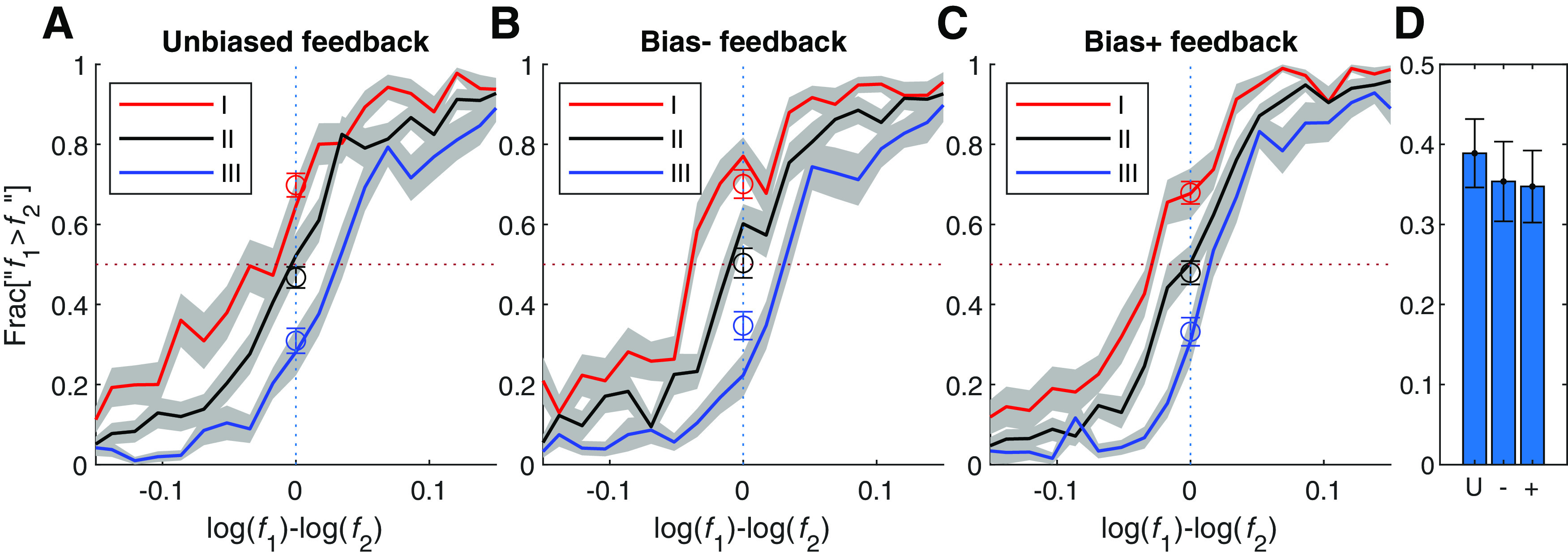
The feedback protocol did not affect contraction bias. ***A–C***, The psychometric curves calculated separately for each of three stimulus ranges (Fig. 1, right), low-frequency range (I) where participants tended to respond “$f_{1}>f_{2}$” (red), high frequency range (III) where participants tended to respond “$f_{1}<f_{2}$” (blue), and an intermediate range (II) evenly distributed around the mean frequency where participants showed no substantial contraction bias (black). These separate psychometric curves were plotted for each of three groups. The psychometric curves were constructed using only the possible trials. ***A***, Unbiased feedback. ***B***, feedback aimed at reducing the contraction bias. ***C***, Feedback aimed at enhancing the contraction bias. ***D***, The bias magnitude, quantified as the distance between the two extreme psychometric curves (at $f_{1}=f_{2}$), was comparable across the three groups (U, unbiased; –, bias –; +, bias +), indicating that the feedback protocol had no substantial effect on the contraction bias. Open symbols in ***A–C*** denote responses in impossible trials. Namely, psychometric curves, based only on possible trials, and the superimposed open symbols were calculated from non-overlapping data points. The near overlap of the two calculations indicates a unified performance with respect to both trial types. Error bars denote the cross-participant SEM.

As explained above, contraction bias is manifested in the tendency to respond “$f_{1}>f_{2}$” when both tones are below the median, and “$f_{1}<f_{2}$” when both tones are above it, as depicted by the shallower slope of the line of indifference plotted in the $f_{1}\times f_{2}$ plane of Figure 1, bottom, right. In order to capture this tendency in the psychometric curves, we divided the trials according to their locations in the $f_{1}\times f_{2}$ plane into three groups (Fig. 1, bottom, right) and plotted the psychometric curves separately for each group of trials (Fig. 1, top, right). Figure 3*A* depicts these three psychometric curves for the unbiased protocol participants. As predicted from Figure 1, top right, because of the contraction bias, the psychometric curve for trials in which $f_{1}$ and $f_{2}$ were small relative to the median (Fig. 1, Region I) is shifted to the left, whereas the psychometric curve for trials in which $f_{1}$ and $f_{2}$ were large relative the median (Fig. 1, Region III) shifted to the right. Therefore, the difference between the fractions of reports “$f_{1}>f_{2}$” when $f_{1}=f_{2}$ is a measure of the magnitude of the contraction bias (Fig. 3*D*, left).

Figure 3*B*,*C* depict the three psychometric curves for the groups of participants administered feedback aimed at reducing and enhancing the contraction bias, respectively. Bias suppressing protocol (bias –) did not decrease the contraction bias relative to the bias enhancing protocol (bias +), indicating that unlike choice bias, the contraction bias was not sensitive to the feedback protocol.

### The impact of feedback protocol on a Perceptron model

**Figure 4.**
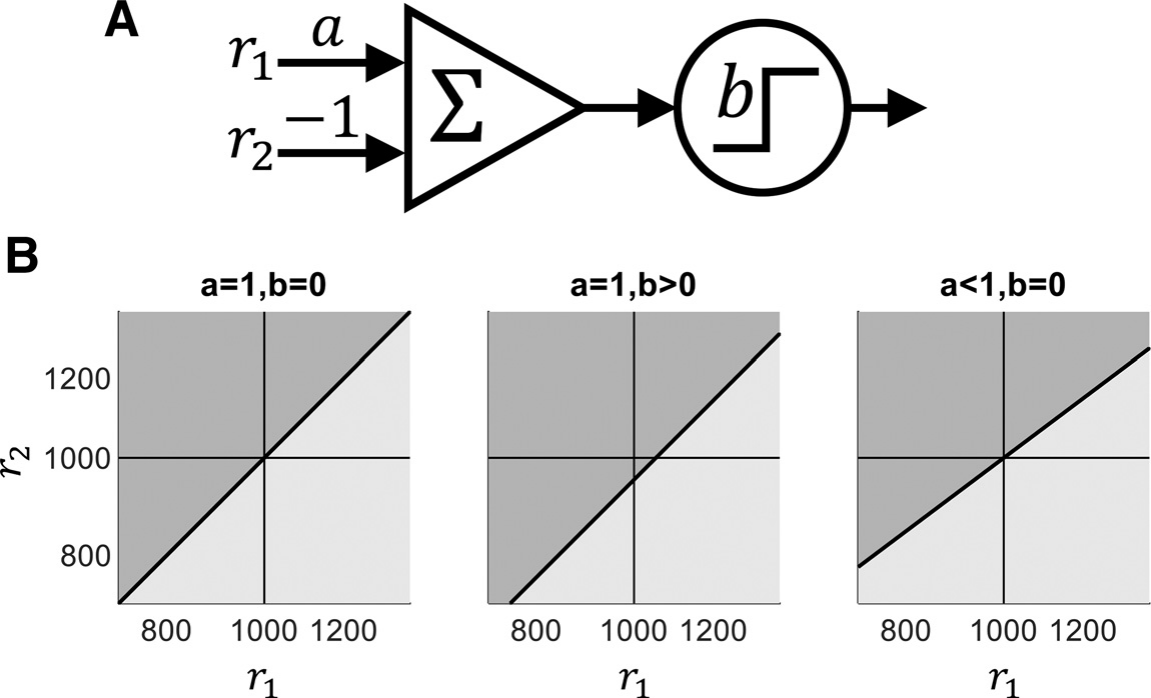
The Perceptron model. ***A***, Schematic illustration of Equation 2. The Perceptron model receives two inputs, $r_{1}$ and $r_{2}$ and responds “$f_{1}>f_{2}$” if and only if $a\cdot r_{1}-r_{2}$ is larger than a threshold b. a affects the first stage in the classification process and determines the slope of the segmentation line in the $r_{1}\times r_{2}$ plane and hence the magnitude of the contraction bias. b affects the decision stage in the classification process and determines the intercept of the segmentation line and thus the magnitude of the choice bias. ***B***, Classification patterns in the$r_{1}\times r_{2}$ plane of different Perceptrons: (left) an unbiased Perceptron with a=1; b=0; center, a=1; b=0.1 resulting in a Perceptron that exhibits choice bias; (right) a=0.75; b=0, resulting in a Perceptron that exhibits contraction bias. Light and dark gray denote “$f_{1}>f_{2}$” and “$f_{1}<f_{2}$” responses, respectively. The behaviors of the Perceptrons in ***B*** are depicted in Figure 1 in the same order.

Could the failure of the feedback protocols aimed to modify the magnitudes of the contraction bias stem from the characteristics of the protocol itself? Our protocols only modified the rewarded responses (“correct” answers) on the impossible trials. One possibility is that optimal adaptation to these protocols does not entail any substantial change in the magnitude of the contraction bias. To address this question quantitatively, we examined the impact of these protocols on binary classification in the framework of the Perceptron model (Rosenblatt, 1958). The Perceptron model is a linear classifier that is consistent with a large family of cognitively and biologically plausible classification schemes. In our application to this task, it compared a noisy representation of the first stimulus with the representation of the second stimulus, as illustrated in Figure 4*A*. Computationally, it models a two-stage hierarchical process in which the representations of the two stimuli ($r_{1}$ and $r_{2}$, respectively) are first linearly combined, after which this combined representation is compared with a threshold. In this simple two-stage architecture, contraction bias stems from the first stage and choice bias results from the second. This framework allowed us to examine both qualitatively and quantitatively, how a feedback protocol is likely to affect each of these biases, and compare it to the actual performance of the human participants in the experiment.

Denoting by $r_{1}$ and $r_{2}$ the representations of the frequencies of the first and second tones in a trial, respectively, we posited that

(1) $$r_{1}=s_{1}+n;r_{2}=s_{2}$$

where $s_{1}$ and $s_{2}$ denote $f_{1}$ and $f_{2}$, measured relative to 1000 Hz (the median of the distribution of the stimuli), This assumption is made for mathematical convenience and as discussed below, does not affect our analysis. All frequencies are measured in the natural logarithm of the frequencies: $s_{i}=\log\left(f_{i}/1000\right)$; n denotes Gaussian noise such that $〈\langle n\rangle 〉=0$ and $〈{\langle n}^{2}\rangle 〉=\sigma^{2}$, where 〈 … 〉 denotes average. This framework assumes that the noise in the task is dominated by noise in the internal representation of the first tone. This asymmetry between the two tones reflects the fact that by the time the second tone is presented and the decision is made, the representation of the first tone is corrupted by the encoding of the first tone in memory and its retention (Ashourian and Loewenstein, 2011). However, the results described below remain qualitatively similar even if noise is assumed to corrupt the representation of the second tone as well, as long as the noise associated with the representation of the first tone is larger than that of the second tone.

Geometrically, the discrimination task is a segmentation of the $r_{1}\times r_{2}$ plane into two regions that correspond to the two possible responses (Fig. 4*B*). Mathematically, all linear classifiers can be implemented by the Perceptron model (Fig. 4*A*):

(2) $$A=\Theta \left(h\right);h=a\cdot r_{1}-r_{2}-b$$

where $\Theta \left(x\right)$ is the Heaviside step function such that $\Theta \left(x<0\right)=0$ and $\Theta \left(x>0\right)=1$, and a and b are parameters. The value of A denotes the response on a trial: A=1 corresponds to reporting “$f_{1}>f_{2}$”, and A=0 corresponds to the opposite response “$f_{1}<f_{2}$”. Figure 4*B* illustrates three different segmentations of the $r_{1}\times r_{2}$ plane, where the dark gray regions indicate the “$f_{1}<f_{2}$” response, and the light gray regions indicate “$f_{1}>f_{2}$” response. Figure 4*B*, left panel, corresponds to the case of a=1 and b=0. It divides this plane according to the sign of $r_{1}-r_{2}$ such that the first and second tones are considered “higher” when $r_{1}>r_{2}$ and $r_{1}<r_{2}$, respectively. This segmentation is clearly optimal in the absence of noise (σ=0) because it corresponds to the segmentation according to the sign of the difference between the two stimuli. The segmentation depicted in Figure 4*B*, middle panel, corresponds to a classifier, which reports that the frequency of the second tone is higher than that of the first tone if and only if $r_{1}-r_{2}>b$ (where b > 0), as illustrated in the vertical shift (downwards when b > 0) of the segmentation line. Figure 4*B*, right panel, depicts a segmentation that is not based on the difference between $r_{1}$ and $r_{2}$. Rather, it is based on a linear combination of $r_{1}$ and $r_{2}$ that weighs $r_{2}$ more than $r_{1},$ i.e., a<1, is manifested in a segmentation line whose slope is less than 1.

Figure 4 depicts the behavior of the model in the space of internal representations $r_{1}\times r_{2}$. To relate it to the experiment, we need to examine its behavior in the $f_{1}\times f_{2}$ plane. To do so, for every pair of stimuli $f_{1},f_{2}$, we computed the probability of responding “$f_{1}>f_{2}$” as a function of the distribution of the noise n and the parameters of the Perceptron. The expected behavior of the three Perceptrons in Figure 4*B* are depicted in Figure 1. When a=1 and b=0 (Figs. 1, 4, left), the model exhibits neither contraction bias nor choice bias; b≠0 (Figs. 1, 4, center) manifested as choice bias and a < 1 results in contraction bias (Figs. 1, 4, right). Thus, the two parameters of the Perceptron b and a naturally map to the two types of bias, i.e., choice bias and contraction bias, respectively.

### The optimal Perceptron model

**Figure 5.**
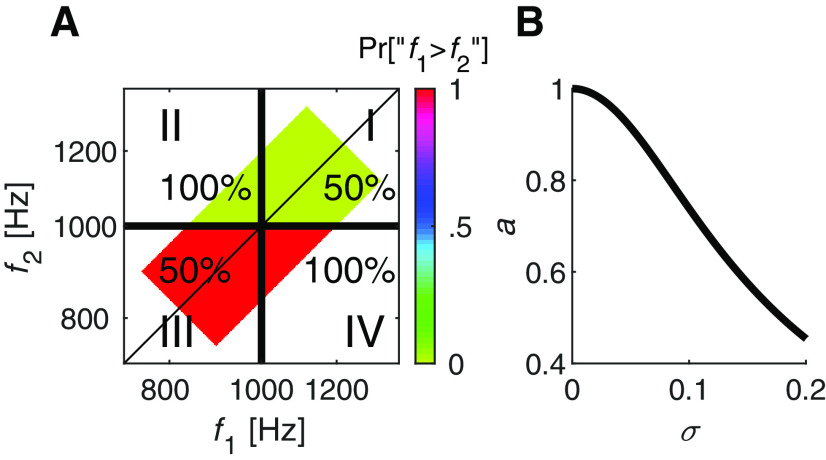
The optimal Perceptron model. ***A***, Performance of a Perceptron with a=b=0. Percentages denote the fraction of correct responses in each quadrant. Despite the fact that the Perceptron ignores the first stimulus in its decision, its performance level is above chance. ***B***, Optimal weighting of the first stimulus, a, as a function of the level of noise σ. Only when σ=0, a=1. The larger the σ the smaller the value of a.

As mentioned above, in the absence of noise (σ=0), the parameters a and b that maximize the performance of the Perceptron are a=1 and b=0, because this type of Perceptron accurately reports which frequency is higher for any pair of frequencies, $f_{1}$ and $f_{2}$. However, in the presence of noise (σ > 0), the value of a that maximizes performance is <1. To see this intuitively, consider the case of infinitely large noise (σ=∞). In this case, the difference between the two represented frequencies, $r_{1}-r_{2}$ is dominated by noise and a classification based on this difference would result in chance level performance. By contrast, consider discrimination by a Perceptron that is characterized by a=b=0. This type of Perceptron, illustrated in Figure 5*A*, which allocates zero weight to the representation of the first tone, would choose its response according to the value of $f_{2}$: it would report “$f_{1}>f_{2}$” (red) when $f_{2}<$1000 Hz and “$f_{1}<f_{2}$” when $f_{2}>$1000 Hz. To see why such classification would result in above-chance performance, consider the performance in the four quadrants. Because of the distribution of stimuli, $f_{1}$ is equally likely to be larger and smaller than $f_{2}$ in Quadrants I (in which this Perceptron reports “$f_{1}<f_{2}$”) and III (in which the Perceptron reports “$f_{1}>f_{2}$”). Therefore, the performance of this Perceptron in these two quadrants will be at chance level. By contrast, in Quadrant II in which $f_{1}<f_{2}$ in all trials and in Quadrant IV in which $f_{1}>f_{2}$ in all trials, the Perceptron would give the correct answer in 100% of the trials. As a result, the overall performance of this Perceptron is better than the chance level performance of a Perceptron that compares the two tones by equally weighing their representations.

More generally, underweighting the first stimulus relative to the second stimulus, which leads to the contraction bias, is beneficial to performance in the presence of noise. From a Bayesian perspective, if the representation of the first stimulus is noisy, the decision maker can benefit by partially replacing that stimulus with information about its prior distribution. Because the representations of the two stimuli are measured relative the median distribution, a Perceptron with 0<a<1 approximately implements this optimal computation. In Materials and Methods, we present an analytical derivation of the optimal value of a for the case of a normal distribution of the first stimulus. Figure 5*B* depicts the optimal value of a as a function of the level of noise σ, the noise in the internal representation of the first tone (note that we assume that there is no noise in the representation of the second tone). The larger the value of σ, the smaller the value of a that optimizes performance (Ashourian and Loewenstein, 2011; Jaffe-Dax et al., 2015) and the larger the contraction bias.

We define an optimal Perceptron to be the Perceptron whose parameters a and b optimize performance given the internal noise σ and the distribution of stimuli. When the feedback is unbiased, the optimal value of b in our model is b=0 for all values of σ. This result is a direct consequence of the fact that we assumed that the stimuli are measured relative to the median of the distribution (1000 Hz; Eq. 1). Because the Perceptron's decision is based on a linear combination of neural activities, the performance of the optimal Perceptron is independent of the baseline used. A different baseline will result in a different optimal value of b which will compensate for the deviation of the baseline from the median of the distribution.

### The optimal Perceptron and human behavior

To compare the optimal Perceptron to the behavior of the human participants, it is worthwhile noting that the optimal Perceptron is characterized solely by a single parameter, namely, the level of noise σ. This is because for every level of noise σ, the values of a and b are uniquely determined by the distribution of the stimuli. In that sense, the complexity of the optimal Perceptron model is identical to that of the classical psychometric curve, which posits that the probability of choice depends solely on the difference between the two stimuli. An unbiased psychometric curve is also characterized by a single number, its width. Mathematically, an unbiased psychometric curve corresponds to a Perceptron with a=1 and b=0.

**Figure 6.**
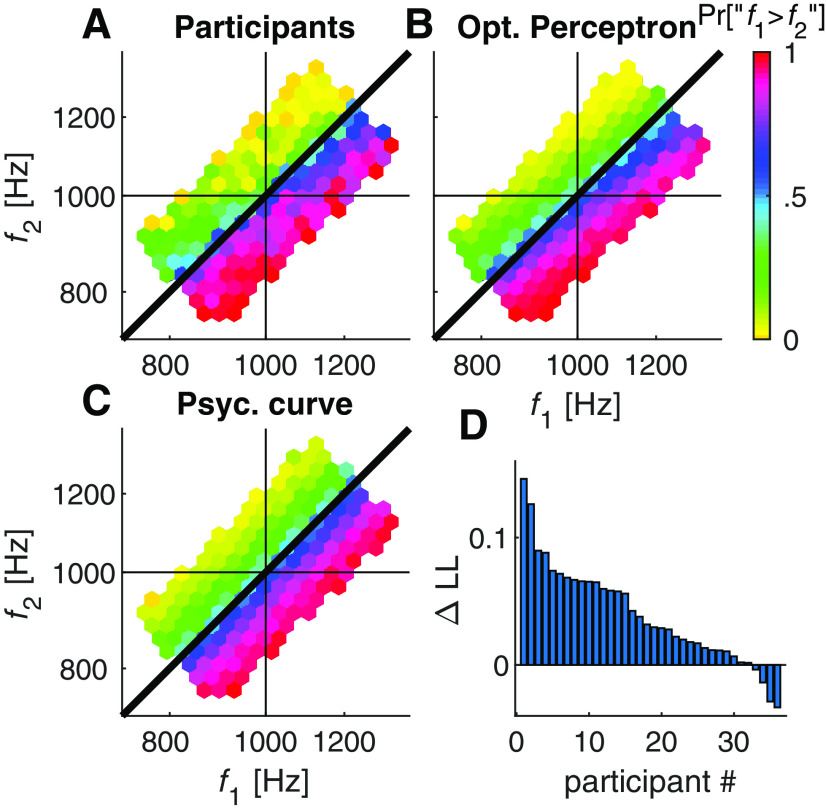
Participants' response and the different models. ***A***, Responses of the human participant. ***B***, responses of the fitted optimal Perceptrons. ***C***, responses of the fitted psychometric curves in the unbiased-feedback protocol. Note that the optimal Perceptron but not the psychometric curve captured human's contraction bias, which was manifested as a shallower line of indifference (iso-color). ***D***, Model comparison. The difference in the log-likelihoods (ΔLL per trial) of the participants' choices for the optimal Perceptron and psychometric curve models across participants. Note that for 89% (32/36) of the participants, the likelihood of the optimal Peceptron model was higher than that of the psychometric curve (both models are characterized by a single parameter).

For each participant in the unbiased feedback protocol (Fig. 6*A*), we used the method of maximum-likelihood to find the value of σ that best fit her behavior according to the optimal Perceptron model (Fig. 6*B*) and the psychometric curve (Fig. 6*C*) models. As illustrated in Figure 6*A–C*, similar to the psychometric curve model, the optimal Perceptron captures the increased accuracy by the distance from the diagonal (change in color in all three plots). By contrast, the optimal Perceptron model also accounts for the contraction bias. Specifically, the line of indifference (equal color, blue) lies along the diagonal in the psychometric curve (which only takes into consideration the difference in frequencies in logarithmic scale); the slope of the line of indifference in the optimal Perceptron model is smaller than 1.

To further compare the psychometric curve and the optimal Perceptron models, we computed for each participant the log-likelihoods of the two models. Because both models are characterized by a single parameter, the level of internal noise σ, their log-likelihoods can be compared directly. Figure 6*D* depicts the difference in the log-likelihood (per trial) of the optimal Perceptron and the psychometric curve models. In each model, the level of internal noise σ was chosen as the value that maximized the likelihood of the model. The fit of the Perceptron model was better for 89% of the participants (32/36, $p<10^{-5}$, binomial test). The difference was particularly pronounced for participants characterized by a larger level of internal noise, for which the Perceptron model predicted a larger contraction bias.

### The impact of feedback protocols on the choice and contraction biases in the optimal Perceptron model

As shown in Figure 6, when the feedback protocol is unbiased, the optimal Perceptron model accounts better for the performance of the human participants than the psychometric curve model. Specifically, the model accounts for the experimentally-observed contraction bias. We used the optimal Perceptron to estimate the expected effects of the different feedback protocols on the choice and contraction biases. To that end, we considered the expected responses of Perceptrons whose parameters a and b are optimized to maximize performance in each of the different feedback protocols. As an estimate of the population distribution of σs, we used the values of σ of the different participants in the unbiased feedback protocol, estimated using the optimal Perceptron model. For each value of σ and for each feedback protocol we computed the values of a and b that maximized the success rate for that protocol. Note that because the parameters of the optimal Perceptron are independent of any particular learning algorithm, we did not explicitly model the learning of these parameters. We then computed the expected performance of these optimal Perceptron using the same pairs of $f_{1}$ and $f_{2}$ as in the experiment. Finally, for each feedback protocol, we averaged the response probabilities of the different optimal Perceptrons.

**Figure 7.**
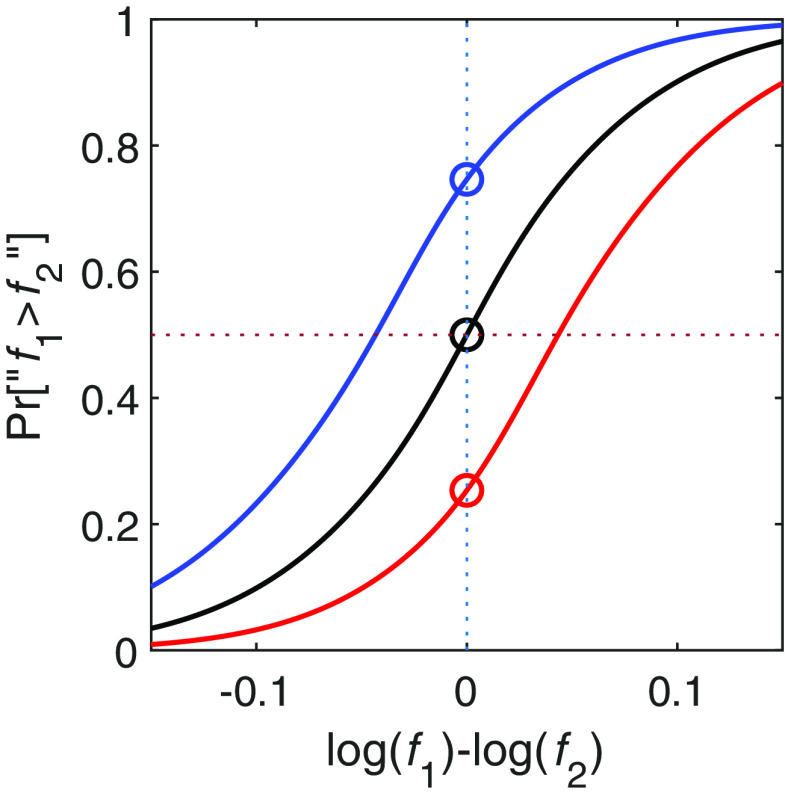
Choice bias and the optimal Perceptron model. The psychometric curves of the optimal Perceptron models whose parameters were optimized to the different reward protocols. Black, unbiased feedback protocol, yielding no choice bias; blue, in the protocol that rewards “$f_{1}>f_{2}$” responses on 90% of the impossible trials; red, in the protocol that rewards “$f_{1}<f_{2}$” in 90% of the impossible trials.

Figure 7 depicts the behavior of the optimal Perceptrons for the feedback protocols favoring “$f_{1}>f_{2}$” response (blue), feedback favoring “$f_{2}>f_{1}$” (red) and unbiased feedback (black). We found that the optimal Perceptron was sensitive to this manipulation, similar to participants' sensitivity. Specifically, the rewarding “$f_{1}>f_{2}$” resulted in a psychometric curve that shifted to the left, whereas rewarding “$f_{1}<f_{2}$” results in a psychometric curve that shifted to the right.

**Figure 8.**
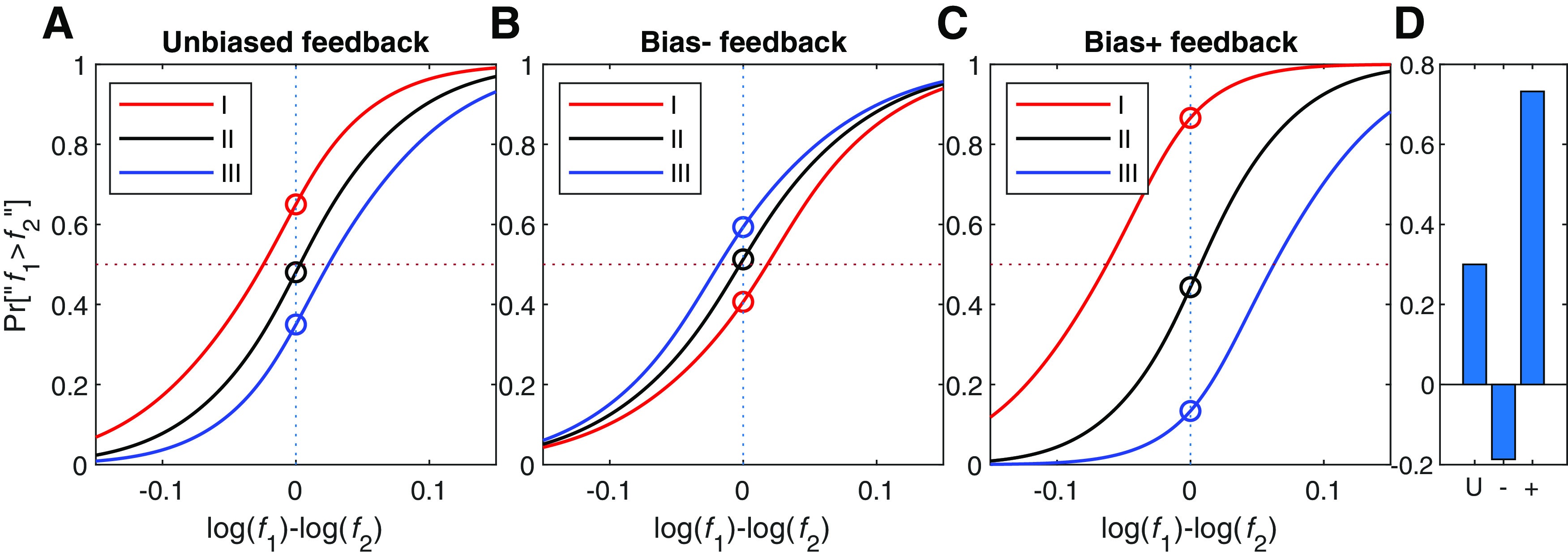
Contraction bias and the optimal Perceptron model. ***A–C***, The psychometric curves of the optimal Perceptron model were calculated separately for each of three stimulus ranges (Fig. 1, right), low-frequency range (I) where participants tended to respond “$f_{1}>f_{2}$” (red), high frequency range (III) where participants tended to respond “$f_{1}<f_{2}$” (red), and an intermediate range (II) evenly distributed around the mean frequency, where participants showed no substantial contraction bias (black). These separate psychometric curves were plotted for each of three groups of optimal Perceptrons whose parameters (a and b) were optimized for (***A***) the unbiased feedback protocol, (***B***) feedback aimed at reducing the contraction bias, and (***C***) feedback aimed at enhancing the contraction bias. ***D***, The bias magnitude, quantified as the distance between the two extreme psychometric curves at $f_{1}=f_{2}$, indicates that the optimal Perceptrons were sensitive to feedback protocol. Open symbols denote responses in impossible trials.

To test the effect of the feedback protocols on the contraction bias of the optimal Perceptron, we administered the two feedback protocols that we administered to our human participants, which were designed to modify the contraction bias. The results are depicted in Figure 8. Whereas for the unbiased-feedback protocol, the optimal Perceptron model predicted psychometric curves in the three frequency ranges comparable to those observed in the human participants (compare Figs. 8*A* and 3*A*), it also predicted that that the bias + and bias – reward protocols would substantially affect the bias, reversing the order of psychometric curves in the bias – condition (Fig. 8*B*) and doubling it in the bias + condition (Fig. 8*C*; see also Fig. 8*D*). This sensitivity of the optimal Perceptron's contraction bias to the feedback manipulations contrasts sharply with the performance of our participants, who did not exhibit any sensitivity to these feedback manipulations.

In the framework of the Perceptron model, our results demonstrate that whereas the parameter b in the Perceptron model is readily modifiable by the feedback protocol, the parameter a is insensitive to these manipulations, at least for the number of trials used in our experiment.

## Discussion

Learning is the process of using experience to gain expertise. In the field of machine learning, it is common to characterize the learning according to the nature of interaction between the learner and the environment. In supervised learning, the learner utilizes information during the training procedure (experience) that it lacks when its expertise is tested, whereas in unsupervised learning, there is no difference between training and test data (Shalev-Shwartz and Ben-David, 2014). Applying this distinction to our task, the extent to which participants use the feedback defines whether learning is supervised or unsupervised.

Behavioral data are well fit by the optimal Perceptron model. Conceptually, the performance of any reinforcement learning algorithm that optimizes the parameters of the Perceptron based on feedback (Mongillo et al., 2014); would result in an optimal-Perceptron like behavior, and specifically, would exhibit contraction bias (Barak et al., 2013). Therefore, one could expect that contraction bias would be sensitive to feedback. However, this is not the case in human behavior. The insensitivity of the contraction bias to the feedback in our human experiments suggests that contraction bias is not a special case of optimization via reinforcement learning. Rather, unsupervised learning underlies the first stage of the computation in human delayed discrimination (Ashourian and Loewenstein, 2011).

### The role of feedback in delayed discrimination

The impact of feedback on choice bias in human perception has been evaluated in several previous studies, most notably with Vernier tasks, where participants are required to report the direction of misalignment between two simultaneously-appearing parallel lines. Wrong (reverse) feedback in a subset of particularly difficult trials has been shown to change the decision criterion (threshold) not only in those trials, but also induce a choice bias on less difficult trials (Herzog and Fahle, 1999). These results are consistent with our findings that manipulating the feedback in a subset of the trials (impossible trials) can affect the decision criterion in the other (possible) trials. In another interesting study, the feedback to Vernier stimuli in different spatial positions was biased in opposite directions and participants developed opposite choice biases at these positions (Herzog et al., 2006). The analogous experiment in our context would be to induce opposite choice biases for different frequencies by providing opposite biased feedback in impossible trials with different frequencies.

Perceptual discrimination and learning in Vernier tasks has been modeled as a two-stage process (Petrov et al., 2005; Liu et al., 2014). The first stage extracts the relevant features from the stimuli; in the second stage, a decision is made by comparing the linear combination of these features to a decision threshold. Importantly, the first representation stage in their model is not sensitive to feedback (Petrov et al., 2005). This assumption is consistent with the implications of our finding that the contraction bias is not modifiable by feedback (though learning of external statistics was not incorporated into their model). However, Liu et al. (2014) and Petrov et al. (2005) posited an additional top-down influence on the decision threshold that drives participants to choose both options with equal probabilities (Petrov et al., 2005). Our optimal Perceptron model, whose parameters are optimized to our specific task, does not incorporate this type of term. This term enables integration of participants' long-term priors, like overall symmetry, across tasks.

Note that the process of learning is not explicitly described in the optimal Perceptron model (for the role of feedback in perceptual learning, see Aberg and Herzog, 2012). Rather, it selects the optimal parameters as a function of the task, as manifested in the feedback protocol. By contrast, learning in the Vernier task was explicitly studied under the assumption of a Hebbian learning rule. The advantage of incorporating a particular learning rule is that it allows the modeling of trial-by-trial learning. Relying on optimality considerations (optimal Perceptron), enables us to draw conclusions that are independent of the specific learning rule.

### Statistical learning, an automatic predecision process

To examine whether contraction bias is indeed modified by the pattern of the input, we manipulated the distribution of stimuli and assessed its impact on performers' bias, in two previous studies. In the visual modality (Ashourian and Loewenstein, 2011), participants were asked to determine which of two serially presented bars is longer. We calculated participants' contraction bias from participants' bias in impossible trials, in which both bars were of the same length, and contraction bias could be attributed only to lengths distribution, which was uniform. Consistent with an ideal observer model, who utilizes the prior distribution of stimuli to maximize performance, participants tended to report that the second bar was longer when both bars were long relative to the median of the distribution. The opposite bias was observed when both bars were relatively short. Importantly, when the range of bar lengths used in the experiment shifted, so was their bias. In response to exactly the same pair of bars, participants tended to report that the first one was shorter in a context in which the two bars were relatively long, and to report that the first bar was longer in a context in which they were relatively short. We have also shown a similar pattern of behavior in the auditory modality, in two-tone frequency discrimination (Lieder et al., 2019). Four different frequency distributions were used with four different groups of participants: uniform spanning two frequency octaves, uniform spanning three octaves, Gaussian, and bimodal, with two uniform one-octave modes separated by one octave. Ideal observer's bias functions substantially differ between these distributions. Participants' bias functions did not differ from that of an ideal observer in any of the distributions. Together, these studies show that contraction bias is modified by bottom-up stimuli modifications in a manner that matches that expected from an ideal decision maker.

The observation that contraction bias is feedback insensitive is in line with previous claims that the bias genuinely affects the perceptual experience and precedes the decision stage (Burr and Cicchini, 2014; Fischer and Whitney, 2014; John-Saaltink, et al., 2016); rather than occurring at a postperceptual decision stage (Alais et al., 2017; Fritsche et al., 2017). Despite being an automatically driven process, contraction bias' magnitude may be manipulated indirectly by task-related attention (Fischer and Whitney, 2014). Attention in this case may operate by enhancing the response to the attended stimuli so that the enhanced contraction bias may be a bottom-up effect reflecting larger responses to attended stimuli (Treue, 2004).

### Perceptual discrimination in the brain

Pioneering studies in monkeys performing an analogous delayed-discrimination task using vibrotactile stimuli found that the sequence of processes underlying task performance is implemented hierarchically by a sequence of brain areas. Neurons in the primary sensory cortex, S1, are phase-locked to the stimulus. Further upstream, neurons in S2 use this information to encode the instantaneous frequency of the vibrotactile stimulation via their firing rates. A series of higher level frontal areas maintain a memory trace of the stimulus during the delay period, and use it to compare the two stimuli in the subsequent decision stage (Romo and Salinas, 2003; Machens et al., 2005;). More recent studies have used a similar delayed-discrimination task in rats to compare the magnitudes of two temporally separated whisker stimulations. Similar to the monkey studies, single-neuron activity in the vibrissal sensory cortex (vS1) was modulated by the temporal fluctuations in the speed of the stimulator. This precise information is lost upstream, in the vibrissal motor cortex (vM1), where activity is modulated by the mean speed of the vibration. Moreover, activity in vM1 is more similar to the perceived stimulus than the activity in vS1 (Fassihi et al., 2017; Mongillo and Loewenstein, 2017).

Recent studies revealed that the posterior parietal cortex (PPC) is a critical locus for the representation and use of prior information in the delayed comparison task, in both rats in humans. In rats trained to compare the loudness of two temporally separated pink-noise auditory stimuli, PPC neurons were found to carry more information about previous trial sensory stimuli than about current trial stimuli. Remarkably, inactivation of the PPC substantially reduced the magnitude of the contraction bias. By contrast, this inactivation had no significant effect on non-sensory biases (Akrami et al., 2018). In humans, performing two-tone delayed discrimination with a fixed reference frequency yields fast improvement (Nahum et al., 2010), which is associated with decreased activation in two cortical regions: the expected auditory region (posterior superior-temporal), and the (left) posterior parietal region (Daikhin and Ahissar, 2015) as in rats.

These results suggest that while the contraction bias is associated with activity in the PPC, non-sensory biases, including those introduced by feedback, are associated with other brain regions, and possibly the frontal networks. Our study indicates that cognitively, perceptual discrimination is a two-stage process, in which unsupervised and supervised learning are separated cognitively, and are associated with sensory and non-sensory biases, respectively.
